# Collagen VI and Hyaluronan: The Common Role in Breast Cancer

**DOI:** 10.1155/2014/606458

**Published:** 2014-07-14

**Authors:** Evgenia Karousou, Maria Luisa D'Angelo, Katerina Kouvidi, Davide Vigetti, Manuela Viola, Dragana Nikitovic, Giancarlo De Luca, Alberto Passi

**Affiliations:** ^1^Department of Surgery and Morphological Sciences, University of Insubria, Via J. H. Dunant 5, 21100 Varese, Italy; ^2^Department of Histology-Embryology, University of Crete, 71003 Heraklion, Greece

## Abstract

Collagen VI and hyaluronan are widely distributed extracellular matrix macromolecules that play a crucial role in tissue development and are highly expressed in cancers. Both hyaluronan and collagen VI are upregulated in breast cancer, generating a microenvironment that promotes tumour progression and metastasis. A growing number of studies show that these two molecules are involved in inflammation and angiogenesis by recruiting macrophages and endothelial cells, respectively. Additionally, collagen VI induces epithelial-mesenchymal transition that is correlated to increased synthesis of hyaluronan in mammary cells. Hyaluronan has also a specific role in cellular functions that depends mainly on the size of the polymer, whereas the effect of collagen VI in tumour progression may be the result of the intact molecule or the C5 peptide of *α*3(VI) chain, known as endotrophin. Collectively, these findings strongly support the parallel role of these molecules in tumour progression and suggest that they may be used as prognostic factors for the breast cancer treatment.

## 1. Introduction

The mammary gland consists of a branching ductal system that ends in terminal ducts known as ductal-lobular units (TDLUs) and an interlobular matrix consisting of fat and fibrous tissues that represent the extracellular matrix (ECM) of adipocytes and stromal fibroblasts [1]. Most breast cancers arise in the ductal-lobular units that are characterized by the presence of a basement membrane and luminal, myoepithelial, and progenitor/stem cells [1–3]. Progression of the ductal hyperproliferation involves the* in situ* and invasive carcinoma and finally the metastasis.

ECM is composed of a complex mixture of macromolecules and a variant of secreted molecules, such as growth factors, that through binding to specific ECM molecules and membrane receptors may activate signalling pathways that control or alternate cell behaviours [4]. The principal proteinaceous components of the ECM are collagens that are produced and secreted by a variety of stromal cells and provide much of the scaffold necessary for the organization of cells that constitute the tissue. Collagen VI is widely distributed in the ECM of various tissues, such as skeletal muscle and adiposal tissue, where it forms a discrete network of beaded microfilaments that interact with other ECM molecules and provide structural support for cells [5]. Furthermore, it activates signaling pathways that regulate cellular functions, such as angiogenesis and autophagy [6, 7]. A second class of molecules that play an essential role in the composition of the ECM is proteoglycans (PGs) and especially the polysaccharidic chains, known as glycosaminoglycans (GAGs), which are covalently bound to the protein core [8]. Hyaluronan is the only GAG that is not bound to a protein but has a fundamental role in tissue homeostasis, as it traps high amounts of water. The role of hyaluronan in cell functions depends on its size and the type of specialized glycoprotein receptors present on the cell membrane. The ECM is not a static structure but is constantly being remodelled by proteolytic enzymes such as the matrix metalloproteinases (MMP) or by the cells through internalization and degradation by lysosomal enzymes, as what happens in the case of hyaluronan [9]. All stages of breast cancer are characterized by an alternate hyaluronan size and deposition in breast tissue stroma that enhances the tumour cell proliferation, invasion, and migration.

Taking into consideration that breast cancer is one of the leading causes of cancer related deaths among women, exploring the alterations of ECM of mammary gland and the role in tumour processes is of central importance in cancer biology. In fact, an increase of collagen and fat deposition in the ECM, including the collagen VI secreted by adipocyte cells, indicates a high mammographic density that is correlated to an increased risk to present breast cancer [10]. In this review, we are willing to present the role of two important ECM components in breast cancer progression: hyaluronan and collagen VI. Both molecules are implicated in the regulation of a number of cell and tissue processes, and for this reason it is worth clarifying the molecular mechanism underlying their contribution to breast tumor progression. Here, we are focused on the role of hyaluronan and collagen VI in breast cancer related angiogenesis, inflammation, and epithelial-mesenchymal transition (EMT), as well as the potential use as serum biomarkers in cancer diagnosis.

### 1.1. Hyaluronan Properties and Its Role in Biological Mechanisms

Hyaluronan is unique among the GAG family as it is the only polymer that is nonsulfated and not bound to a protein core. The structure of hyaluronan is characterized by repeating disaccharide units of D-glucuronic acid and N-acetyl-D-glucosamine linked by a glucuronidic *β*(1 → 3) bond. The disaccharide units are then linearly polymerized by hexosaminidic *β*(1 → 4) linkages. The number of repeating disaccharides in a completed hyaluronan molecule can reach 10,000 or more and can reach a molecular weight of 6 to 8 MDa. The average length of a disaccharide is about 1 nm. Thus, a hyaluronan molecule of 10,000 repeats could extend 10 *μ*m if stretched from end to end, a length approximately equal to a diameter of a human erythrocyte.

Hyaluronan is ubiquitously distributed in vertebrates, whereas it has been identified in some of the lower marine organisms and in certain bacteria. It is a main component of all connective tissue and ECM in mammals and is more abundant in the rapidly growing fetal tissues, especially in the first weeks of gestation [11], than in mature adult tissues. Indeed, hyaluronan-rich environment promotes cell proliferation and migration which is important during embryogenesis for the rapid move of stem cells to the site of organ development [12]. On the other hand, the hyaluronan-rich environment actively inhibits differentiation of cells and cells must lose their hyaluronan coat in order to differentiate.

Despite the simple structure of hyaluronan, the importance that is exhibited in biological mechanisms depends on the molecular size. High molecular weight (HMW) hyaluronan forms complexes with other macromolecules of the ECM, regulating the cell adhesion, motility, and growth, whereas hyaluronan oligosaccharides regulate cell behaviours through interaction with cell surface receptors [13]. Thus, the fine regulation of synthesis or degradation to different size of hyaluronan is significant for the physiology of the tissues and is also crucial in malignancies.

Hyaluronan is closely associated with tumourigenesis as it influences tumour cell behaviour and cancer progression by modulating the hydration and osmotic balance in the tumour environment. By interaction with specific receptors, hyaluronan is capable of intracellular signal transduction that can promote the malignant phenotype [14]. Several studies have reported a relation between HA content and invasiveness, as well as a greater enrichment of hyaluronan in the stroma that surrounds tumours than in parenchymal regions. Other studies have shown that hyaluronan production by stromal cells is stimulated by interactions with tumour cells, but that synthesis is also increased in malignant tumour cells themselves. In patients with cancer, hyaluronan concentrations are usually higher in malignant tumours than in corresponding benign or normal tissues, and in some tumour types the level of hyaluronan is predictive of malignancy. However, hyaluronan levels can be increased around tumour cells themselves or within the tumour stroma. In patients with breast and ovarian carcinomas, high levels of hyaluronan in the stroma are associated with low survival rates [12].

### 1.2. Hyaluronan Synthesis and Catabolism: Hyaluronan Synthase (HAS)

Hyaluronan is synthesized by a family of enzymes, called hyaluronan synthases (HASs). In mammals, there are three HAS isoforms (HAS1, HAS2, and HAS3) that are expressed by genes located in different chromosomes and share 55–71% sequence identity [15]. The HAS proteins have molecular masses from 42 to 64 kDa and differences in enzymatic properties particularly in their ability to form hyaluronan matrices and determine product size [16] under the control of a wide variety of cytokines and growth factors. The gene expression of HASs in mammals is tissue and cell specific and varies between normal and pathologic conditions. All HAS proteins are localized within the plasma membrane, containing multiple membrane-spanning regions and large cytoplasmic loops. The catalytic activity of HASs resides in the inner face of membrane with active sites for the two precursors, UDP-glucosamine and UDP-glucuronic acid, and the product is secreted or translocated through the HAS protein complex to the ECM [17].

The changes in hyaluronan synthesis can be related to HAS mRNA expression, to availability of the UDP-sugar precursors, or to posttranslational modifications [18]. In particular, it was demonstrated that, at low ATP/AMP ratios, HAS2 is phosphorylated by the adenosine monophosphate activated protein kinase (AMPK) at threonine 110, which resides in a cytoplasmic loop, resulting in a decreased hyaluronan synthesis [19]. Moreover, HAS2 activity is regulated by oligomerization and monoubiquitination at lysine 190 [20]. Recently, it was shown that HAS2 can also undergo regulation by O-GlcNAcylation that has an important impact on alterations in metabolism, since in hyperglycaemic conditions there is an increase of UDP-GlcNAc that, in turn, leads to an increase of O-GlcNAcylation [21].

In breast cancer, expression of HAS2 is mainly in metaplastic carcinomas of breast, which is a subtype related to the EMT, and less expressed in invasive ductal carcinomas [22]. Moreover, expression levels of stromal HAS1 and HAS2 during breast tumour are related to obesity, large tumor size, lymph node positivity, and estrogen receptor negativity [23]. Both HAS2 and HAS3 synthesize and extrude HMW-hyaluronan, which in invasive cell lines is rapidly depolymerised into fragments ranging from 10 to 40 kDa that are involved in neovascularisation [14]. Suppression of HAS2 in MDA-MB-231 breast cancer cell line displayed decreased cellular proliferation with a transient arrest in a cell cycle, ablation of migratory phenotype, alteration in hyaluronan catabolism, and inhibition of primary and secondary tumour formation [14]. Recently, it was demonstrated that HAS2 expression in NMuMG mammary epithelial cells is required for the TGF-*β*-mediated EMT [24].

### 1.3. Hyaluronan Synthesis and Catabolism: Hyaluronidase (HYAL)

Hyaluronan has a high turnover in the body, as one third is degraded each day. A major part of the circulating hyaluronan is taken up by the liver and a minor part by the kidneys [25]. In joints 20–30% of hyaluronan is catabolised by local degradation. The lymphatic tissue carries HA to the blood stream where 80–90% is degraded by receptor mediated catabolism [26]. The hyaluronan degrading enzymes, known as hyaluronidases (HYALs), hydrolyze predominantly the hexosaminidic *β*(1 → 4) linkages between N-acetyl-D-glucosamine and D-glucuronic acid residues. These enzymes also hydrolyze *β*(1 → 4) glycosidic linkages between N-acetyl-galactosamine or N-acetylgalactosamine sulfate and glucuronic acid in chondroitin and dermatan sulfates [27]. From the expressed sequence tag (EST) database, there are established six such sequences in the human genome. In the human, three genes (HYAL1, HYAL2, and HYAL3) are found tightly clustered on chromosome 3p21.3, coding for hyaluronidases 1, 2, and 3. As in synthesis, in degradation HYAL type expression is cell and tissue specific and expression levels of HYALs vary in different tumours.

Hyaluronan is mainly synthesized as a HMW-hyaluronan and then degraded in oligosaccharides by HYALs. The HMW-hyaluronan in general promotes tissue integrity and quiescence, while hyaluronan breakdown products signal that injury has occurred. The smaller fragments of hyaluronan accompanied by increased HYAL expression are involved in a variety of normal and pathological processes and can either promote or inhibit tumour progression [28]. However, HYAL has been used for therapeutic purposes in breast cancer treatment as it is relatively nontoxic, degrades the matrix hyaluronan, and enhances the penetration of cytostatic drugs [29].

### 1.4. The Role of Hyaluronan Receptors CD44 and RHAMM in Tumour Progression

CD44, a hyaluronan receptor, is a transmembrane glycoprotein with ability to bind HA with high specificity [30]. Importantly, this molecule participates significantly in cell-cell and cell-matrix interactions [31]. It is encoded by a single highly conserved gene but appears in multiple isoforms of different molecular size, ranging from 80 to 200 kDa. These isoforms are generated both by alternative splicing of 20 exons and through protein modifications. The isoform that is mainly expressed in normal cells is an 85 kDa protein that contains none of the variable exons and has been shown to mediate the hyaluronan-promoted motility in breast cancer cell lines [30, 32, 33]. Variant expression is linked to tumour progression and is regulated by tissue and environment-specific factors and signalling pathways involved in oncogenesis including the Ras-MAPK cascade [34]. CD44 is composed of an extracellular domain, which contains binding sites for molecules such as hyaluronan, a membrane region, a transmembrane domain, and a cytoplasmic tail. The latter domain of CD44 recruits regulatory proteins to the cell membrane activating various signalling pathways [35, 36]. It is also involved in the association of signalling complexes with the actin cytoskeleton [31, 37]. In healthy tissues, CD44 plays a fundamental role in the regulation of basic cellular processes such as cell adhesion, inflammation, and repair [38].

RHAMM is a hyaluronan-binding protein/receptor that has a diverse cellular localization including the cell surface, the cytoplasm, and the nucleus and it is also found secreted to the ECM [39]. Its expression in normal tissues is not very high but it is found to be overexpressed in many advanced cancers [33]. Different RHAMM isoforms are produced due to alternative splicing and the expression of these transcript variants is different depending on the type of the cells [40]. RHAMM protein is glycosylphosphatidylinositol-anchored to the cell membrane as it lacks a typical transmembrane domain [41]. At the cell surface, extracellular RHAMM is required for tumour cell motility and invasion, affecting hyaluronan and growth factor-induced MAPK (ERK1, 2) signalling via associations with transmembrane hyaluronan receptors such as CD44 and protein tyrosine kinase (PTK) receptors such as PDGFR and RON [42, 43]. Intracellular RHAMM, on the contrary, regulates crucial processes in cancer cells [44], through its binding to actin filaments, microtubules, podosomes, the centrosome, and the mitotic spindle [33]. Therefore, the role of RHAMM seems to be associated with its expression levels and cellular distribution.

### 1.5. Collagen VI Structure and Biological Role

Collagen type VI is a microfibrillar component of the ECM and has a ubiquitous distribution throughout connective tissues. It plays a key role in the maintenance of tissue integrity by providing a structural link between different components of connective tissues, basement membranes, and cells. Collagen VI is thought to participate in cell adhesion, spreading, and migration of various cell types through interaction with member of the integrin receptor family or the NG2 PGs, contributing directly by this to the regulation of cell behaviour by receptor-mediated signal transduction pathways [45]. Moreover, collagen VI monomers or individual chains can bind to collagen type I and type III, hyaluronan, heparin [46, 47], or interact with other matrix components, such as PGs, suggesting that collagen VI microfibril supramolecular assemblies act as scaffolds for the formation of the structurally critical fibrillar collagen networks [48].

Collagen VI is a heterotrimer composed of three genetically distinct *α*1(VI), *α*2(VI), and *α*3(VI) polypeptide chains [49] encoded by* COL6A1*,* COL6A2*, and* COL6A3* genes, respectively. The intact molecules associate laterally in an antiparallel fashion into dimmers that are stabilized by disulfide bridges [50]. The dimmers aggregate further into tetramers that are secreted into the ECM [51, 52], where they join end to end into microfibrils. Recently, they were characterized three additional chains, the *α*4(VI) that forms trimers with *α*1(VI) and *α*2(VI), the *α*5(VI) and *α*6(VI), with a high degree of similarity with the *α*3(VI) chain, that were found accumulated intracellularly [53].

The *α*1(VI), *α*2(VI), and *α*3(VI) chains of collagen VI contain a short triple-helical region and globular extensions at the N and C terminus. The *α*1(VI) and *α*2(VI) chains are similar in size and structure with Mr 140 kDa, while the third chain *α*3(VI) is much larger with Mr ranging from 180 to 240 kDa. The *α*3(VI) chain has 10 vWF type A domains of approximately 200 amino acids at the N-terminus (N1–N10, N10 being the most N-terminal) compared with only one such domain in the *α*1(VI) and *α*2(VI) chains [54]. Unlike the C1 and C2 domains of all three collagen VI subunit chains, the three additional C-terminal domains of *α*3(VI) chain have no homology to vWF type A domains. The proline-rich C3 domain shows some similarity to salivary proteins, C4 shows homology to type III repeats found in fibronectina and tenascin, and C5 is related to apoprotein-type protease inhibitors [54]. The cleavage of C5 domain, called also endotrophin (ETP), occurs immediately after the secretion of collagen VI. Nothing is known about the protease that cleaves the ETP-peptide. However, recent studies have demonstrated that matrix metalloproteinase-11 (MMP11)/stromelysin-3, a zinc endopeptidase belonging to the MMP family, cleaves collagen VI and in particular the native *α*3 chain of collagen VI [55]. Moreover, it has been demonstrated in* in vivo* experiments that the absence of MMP11 in deficient mice dramatically alters the collagen VI folding and subsequently adipocytes and related ECM [55]. Additionally, the ectopic expression of MMP11 at the adipocyte-cancer cell interface leads to collagen VI alteration [55].

Missense and homozygous or heterozygous mutations of the genes expressing the three chains have been shown to cause Ullrich congenital muscular dystrophy and Bethlem myopathy, genetic diseases characterized by muscle weakness [56]. In addition, overexpression of* COL6A1* and* COL6A2* and higher synthesis of collagen VI have been observed in the umbilical cord and the ECM of the skin fibroblasts from foetuses and individuals, respectively, affected with trisomy 21 [57, 58]. As it will be discussed below, alterations in collagen VI and especially ETP peptide demonstrate a crucial role in breast cancer progression [59, 60]. Thus, abnormalities in collagen VI can lead to a wide spectrum of clinical phenotypes and pathologies.

## 2. Correlations between Collagen VI and Hyaluronan

Hyaluronan and collagen VI are two components of the ECM that interact with each other [46, 60]. In detail, it has been reported that the ECM of human articular cartilage comprises a network of collagen VI that interacts with PGs and hyaluronan [60]. Moreover, interaction of collagen VI and hyaluronan was also demonstrated in* in vitro* studies [46, 47, 61] and in hyaluronan-based polymer scaffolds in tissue engineered cartilage [62]. Recently, we demonstrated in our study that overexpression of collagen VI induced HAS2 gene expression and hyaluronan synthesis. Even though there are no data regarding a direct crosstalk between hyaluronan and collagen VI in breast cancer, they both promote tumour progression via common pathways. In this review, we summarize and discuss data that demonstrate the common role of collagen VI and hyaluronan in inflammation, angiogenesis, and EMT in breast cancer.

## 3. Collagen VI and Hyaluronan in Breast Cancer

### 3.1. Localization* In Vivo* and Synthesis* In Vitro*

An important factor for the breast cancer cell behaviour is the presence or absence of the estrogen receptor (ER) within the cell membrane. Clinically, ER-positive breast cancer is less aggressive than the ER-negative and is amenable to hormone therapy by ER modulators. In basic research several breast cancer cell lines have been used for the* in vitro* study of breast cancer, taking into consideration additionally the progesterone receptor (PR) and the human epidermal growth factor receptor 2 (HER2). MCF-7 and MDA-MB-231 are two representative breast cancer lines commonly used in breast cancer research. MCF-7 is an ER+/PR+/HER2-cell line, whereas MDA-MB-231 is an ER−/PR−/HER2-cell line that belongs to the triple negative breast cancer cell lines [63].

Collagen VI is found in the invasive front of the tumour that was defined as the three to six layers of tumour cells at the front edge or the scattered tumour groups between the tumour and the host tissue or organ. The ETP peptide is localized close to adipocytes within the breast tumour, which are the most representative cytotypes in breast tissues and they secrete considerable amount of collagen VI. Regarding hyaluronan, the high amount in breast cancer is observed in the immediate peritumoral stroma or associated with carcinoma cells [64]. The intensity of hyaluronan signal within the breast tumour was significantly related to poor differentiation of the tumours, axillary lymph node positivity, and short overall survival of the patients [65].

Studies performed in rodents and confirmed in human MCF-7 cell line have demonstrated that collagen VI, produced by adipocytes, promotes growth of cancer cell proliferation via NG2/chondroitin sulphate proteoglycan receptor expressed on the surface of malignant ductal epithelial cells and sequentially activates Akt and *β*-catenin and stabilizes cyclin D1. Interestingly, the domain that appears to be responsible for such effect seems to be the ETP peptide. Therefore, adipocytes play a vital role in defining the ECM environment for normal and tumour-derived ductal epithelial cells and contribute significantly to tumour growth at early stages through secretion and processing of collagen VI [66].

Hyaluronan is mainly produced by the stromal fibroblasts of breast tumour, even though both MDA-MB-231 and MCF-7 synthesize hyaluronan in lower amounts [67, 68]. Recent studies demonstrated that the more aggressive the breast cancer cell, the more hyaluronan produce. Indeed, the clone of MDA-MB-231 cell line that forms bone metastases (MDA-MB-231-BM) showed an abundant expression of HAS2 that was higher with respect to wild-type and a more invasive phenotype. The increased HAS2 expression in the MDA-MB-231-BM was correlated to a suppression of TIMP-1 expression, which presumably increased MMP activity with a consequent basement membrane degradation [69].

### 3.2. The Role of Collagen VI and Hyaluronan in Angiogenesis

Tumour angiogenesis is the proliferation of a blood vessel network within a growing malignant tissue and is an essential process for supplying rapidly nutrients and oxygen and removing waste products. An angiogenic switch allows tumour cells to survive and grow and enhances the tumour metastasis. The neovascularisation is influenced by some ECM proteins of the tumour environment [70, 71].

Expression of collagen VI has been found within or close to the blood vessels in juvenile angiofibromas and human glioblastomas.* In vitro* studies demonstrated that endothelial cells incubated with conditioned media from ETP-overexpressing HEK-293T cells stimulated their recruitment, as well as the vessel formation [6]. Thus, ETP peptide acts as chemoattractant that recruits endothelial cells and it was found also that it stimulates angiogenesis by upregulating proangiogenic factors [6, 59].

The role of hyaluronan in angiogenesis depends on the molecular weight of the polymer. Both angiogenesis and intracellular signalling are affected by the degradation products of hyaluronan. The most expressed hyaluronidase in breast cancer cells is HYAL-2, even though recent studies showed that HYAL-1 has a pivotal role in tumour cell behaviour and angiogenesis. Indeed, knockdown of HYAL1 expression in MDA-MB-231 and MCF-7 cells results in decreased cell growth, adhesion, invasion, and angiogenesis potential. In addition, the HYAL1 knockdown showed an inhibition of breast cancer cell xenograft tumor growth and microvessel density, whereas HYAL-1 overexpression demonstrated an increase of tumour growth [72, 73]. A number of reports demonstrate that also PH-20 or SPAM1, which is thought to be a testicular hyaluronidase, is a tumour marker for breast and laryngeal cancer. In fact, PH-20 expression levels are increased in metastatic breast cancer to lymph nodes compared to ductal carcinoma* in situ* and invasive breast cancer [74]. Moreover, the upregulated PH-20 in breast cancer cells MDA-MB-231 was found to promote tumour growth when implanted into the chorioallantoic membrane (CAM) of chicken embryo to form a tumour. The growth was accompanied by an increment of neogenetic vessels and the release of FGF-2 from tumour cells indicating that upregulation of PH-20 in malignant breast tissue may degrade hyaluronan into small fragments and contribute to angiogenesis [75, 76].

The HMW native hyaluronan has been shown to be antiangiogenic by inhibiting endothelial cell proliferation and migration and capillary formation in three-dimensional matrix [77]. Administration of hyaluronan-versican aggregates showed, however, promotion of infiltration of endothelial cells within matrigel plugs containing angiogenic factors, suggesting the stimulation of HMW-hyaluronan in angiogenesis [78]. Thus, the role of the large polymer of hyaluronan in angiogenesis needs further clarification and study.

### 3.3. Collagen VI and Hyaluronan Induce Inflammation

Tumour-associated macrophages (TAMs) are a cell type with potent immunosuppressive functions that favour tumour angiogenesis, growth, and metastasis. TAMs are a polarized M2 macrophage population with similar functions to M2s and have an IL-10^high^ IL-12^low^ phenotype [79]. The predominant expression of these M2 macrophages is associated with more aggressive histopathological features (high tumour grade), increased microvessel density, and decreased overall survival that is the late stage of tumour progression. On the other hand, in advanced stages, TAMs release cytokines such as transforming growth factors *β*1 (TGF *β*1) and IL-10 and promote tumour development through inhibition of anticancer immune responses [80, 81].

The M2 phenotype macrophages induced by transforming growth factor (TGF-*β*1) and inflammatory factors, such as interleukin- (IL-) 4 and IL-10, produce high amount of collagen VI.* In vitro* studies showed also that macrophages use collagen VI to modulate their binding properties rather than to build native ECM as do other cell types, including fibroblasts and smooth muscle cells (SMC), suggesting TAMs as one of the key providers for collagen VI in tumours. [82]. Similarly to collagen VI, the ETP peptide is able to promote tumour inflammation by increasing macrophage recruitment and upregulating the production of inflammatory factors, such as IL-6 and tumour necrosis factor-alpha (TNF-*α*), which are abrogated by anti-ETP antibodies [6]. These outcomes suggest that the majority of chemoattractant properties exerted by collagen VI are performed by ETP [59].

The inflammatory effects of hyaluronan, pro- and anti-inflammatory, are correlated to the size of the polymer. Hyaluronan with an average molecular mass <500 kDa is considered as a fragment, although the molecular properties of a 500 kDa fragment and those of a 50 kDa are different [83]. The indicated size of hyaluronan used in a research study usually represents the average molecular weight of a polydisperse distribution. In regulatory T-cells, HMW-hyaluronan with an average molecular weight of ~10^7^ Dalton binds to CD44 and promotes STAT5 signalling pathway which results in a maintenance of the cells and an inhibition of proliferation, indicating HMW-hyaluronan as an anti-inflammatory agent [84]. On the other hand, hyaluronan fragments of 200 kDa have been shown to stimulate chemokines, cytokines, growth factors, proteases, and nitric oxide by macrophages [83].

Hyaluronan degradation into fragments is performed by hyaluronidase, indicating the important role of the degrading enzyme expression in tumour environment. However, it is of high importance also to consider the role of HAS2 and the synthesis of hyaluronan by stromal fibroblasts in tumour malignancies. Indeed, hyaluronan-rich environment in tumour indicates active migration and proliferation of tumour cells. Moreover, the hyaluronan species participate in TAM trafficking into tumour masses, enhancing the neovascularisation. Recent studies on Has2 null fibroblasts showed severe impairment in recruiting macrophages when inoculated with tumour cells into nude mice, which demonstrates the contribution of stroma-derived hyaluronan in intratumoral macrophage mobilization [85]. Importantly, the upregulation of HAS2 in highly metastatic breast cancer stem-like cells showed its critical role for the interaction of the cells with TAMs, leading to enhanced secretion of platelet-derived growth factor-BB from TAMs, which then activated stromal cells and enhanced the breast cancer stem-like cells self-renewal [86].

### 3.4. Correlation between Collagen VI or Hyaluronan and EMT

Another important factor for tumorigenesis and tumour progression is the epithelial-to-mesenchymal transition (EMT). EMT is characterized by the loss of cell-cell adhesions, destruction of the apicobasal polarity of epithelial cell membranes, and interactions between new plasma membrane receptors and remodelled constituents of ECM [87, 88]. Several studies have used breast cancer in model organisms in order to provide new insights regarding EMT in cancer progression. Both hyaluronan and collagen VI are among the molecules of ECM that induce EMT during cancer progression.

As described above, collagen VI and especially the ETP peptide has a critical role in various events that characterize the growth, invasive, and metastatic capacity of tumours. Recent studies demonstrated that in the ETP transgenic mice (PyMT/ETP) immune responses, cell cycle regulation, and stem cell pluripotency were significantly altered [6]. Notably, a significant decrease of E-cadherin, the loss of which is a characteristic of EMT, and an increased pulmonary metastasis of breast cancer in the PyMT/ETP with respect to the nontransgenic mice were observed [6]. The metastasis was associated with an induction of TGF*β*-dependent EMT by ETP peptides [6]. Therefore, ETP may be considered as one of the ECM molecules that contribute to EMT induction.

TGF*β* potently stimulates hyaluronan synthesis via upregulation of HAS2 in NMuMG [87] and MCF-10A [22]. Specifically, Porsch and colleagues [24] demonstrated that HAS2 expression plays a pivotal role in the TGF*β*-induced EMT, as knockdown of HAS2 in NMuMG cells inhibited the TGF*β*-induced EMT by about 50% and abolished the TGF*β*-induced cell migration. Removal of extracellular hyaluronan by* Streptomyces* hyaluronidase, blocking of CD44 with antibodies, or CD44 knockdown did not inhibit TGF*β*-induced EMT or cell migration, suggesting that HAS2, but not hyaluronan, has a regulatory role in EMT.

Even though nothing is known about a possible cross-linking between hyaluronan and collagen VI or ETP during the EMT process, it is evidently shown that ETP induces TGF*β*-dependent EMT which in turn is regulated by HAS2 expression and results in hyaluronan increase. Therefore, both ETP and HAS2 are essential for the induction and maintenance of EMT in breast cancer and TGF*β* alone is not enough for the EMT process.

### 3.5. Collagen VI and Hyaluronan as Serum Biomarkers

The best available approach for detection of breast cancer currently is the mammographic screening. Although resolution continues to improve, tumours smaller than 5 mm are usually not detectable and mammography effectiveness decreases in young women who physically have dense breast tissue. Several studies are focused on developed techniques for the identification of altered proteins in human serum as an additional approach for the detection, follow-up, and determination of cancer stage of breast cancer [89]. Among them, breast cancer proteins (BC) 1, 2, and 3 are used for the detection and the discrimination of the breast cancer in patients [90].

Serum hyaluronan has been used as a disease marker in pathologies such as liver fibrosis in patients affected with chronic viral hepatitis C and rheumatoid arthritis. Regarding breast cancer detection, serum hyaluronan levels were found significantly elevated in women with severe malignant breast cancer associated with high metastasis, with respect to the nonmetastatic carcinoma or with respect to those with benign breast disease [91]. On the other hand, collagen VI is not considered a serum biomarker of breast cancer, even though the *α*1(VI) chain was among the proteins identified by biopanning from a breast cancer cDNA T7 phage library [92]. Nevertheless, collagen VI is indicated as a diagnostic serum marker of patients with melanoma and pancreatic cancer, since the levels of this protein in human sera are significantly increased in both aggressive malignant tumours with respect to healthy donors.

## 4. Conclusions and Future Perspectives

**Figure 1:**
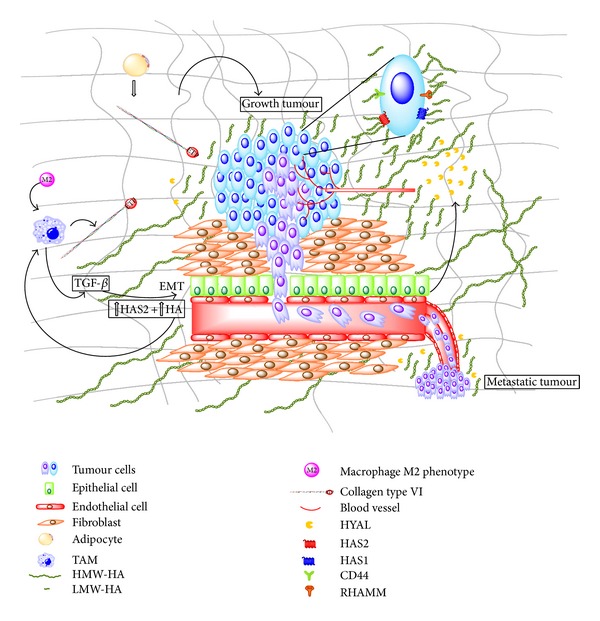
Schematic representation of collagen VI/ETP and hyaluronan contribution in breast cancer progression. Synthesis of collagen VI by adipocytes as well as synthesis of hyaluronan by stromal cells is increased in breast tumour. Macrophages release TGF*β* and collagen VI than in turn increase HAS2 and hyaluronan in mammal cells and induce epithelial-mesenchymal transition (EMT). Increased hyaluronan and cleaved ETP induce recruitment of both macrophages and endothelial cells, resulting in neovascularisation that in turn promotes metastasis. This latter phenomenon is also induced by low molecular weight hyaluronan that is the result of HYAL activity on the HMW molecule. Both collagen VI and HMW-hyaluronan induce growth tumour. Abbreviations: ETP, endotrophin; TGF*β*, transforming growth factor-beta; HMW-HA and LMW-HA: high and low molecular weight hyaluronan, respectively; HYAL: hyaluronidase; HAS: hyaluronan synthase; TAM: tumour-associated macrophages.

As summarized in Figure 1, collagen VI and hyaluronan regulate the breast cancer progression and metastasis and seem to participate in the same biological processes in a synergistic manner. Collagen VI is produced by adipocytes during breast cancer, as well as by macrophages during inflammation. Hyaluronan is mainly produced by stromal cells, even though its increased synthesis by breast cancer cells is correlated to an increased aggressiveness of cancer. Both molecules are responsible for the recruitment of endothelial cells during angiogenesis in the breast cancer microenvironment. Moreover, macrophages produce collagen VI and secrete inflammatory factors that in turn induce hyaluronan synthesis by stromal cells. Both hyaluronan and ETP peptide regulate the trafficking and recruitment of macrophages, demonstrating their synergistic effect in breast cancer progression. Breast cancer is also characterized by EMT process. As discussed above, ETP, hyaluronan, and HAS2 are essential for the induction and maintenance of EMT progression. Thus, simultaneous targeting of these two molecules may be a promising approach for improving pharmaceutical agents and consequently breast cancer treatment.

One of the parameters to take also into consideration is the size of hyaluronan and the amount of intact collagen VI or the ETP peptide during each biological process, which means that the role of HASs, HYALs, and further studies regarding the proteases responsible for the ETP cleavage should be taken into consideration for breast cancer management and prognostication.

## Abbreviations

ECM: Extracellular matrix
HMW: High molecular weight
HYAL: Hyaluronidase
HAS: Hyaluronan synthase
ETP: Endotrophin
TAM: Tumour-associated macrophages
TGF-*β*: Transforming growth factor-beta
IL: Interleukin
EMT: Epithelial-mesenchymal transition
MMP: Matrix metalloproteinase.
